# Effect of cell cycle duration on somatic evolutionary dynamics

**DOI:** 10.1111/eva.12518

**Published:** 2017-10-12

**Authors:** Dominik Wodarz, Ajay Goel, Natalia L. Komarova

**Affiliations:** ^1^ Department of Ecology and Evolutionary Biology University of California Irvine CA USA; ^2^ Department of Mathematics University of California Irvine CA USA; ^3^ Center for Gastroenterological Research Baylor Research Institute and Sammons Cancer Center Baylor University Medical Center Dallas TX USA

**Keywords:** cell cycle delay, checkpoints, damage repair, evolutionary dynamics, Mathematical models

## Abstract

Cellular checkpoints prevent damage and mutation accumulation in tissue cells. DNA repair is one mechanism that can be triggered by checkpoints and involves temporary cell cycle arrest and thus delayed reproduction. Repair‐deficient cells avoid this delay, which has been argued to lead to a selective advantage in the presence of frequent damage. We investigate this hypothesis with stochastic modeling, using mathematical analysis and agent‐based computations. We first model competition between two cell types: a cell population that enters temporary cell cycle arrest, corresponding to repair (referred to as arresting cells), and one that does not enter arrest (referred to as nonarresting cells). Although nonarresting cells are predicted to grow with a faster rate than arresting cells in isolation, this does not translate into a selective advantage in the model. Interestingly, the evolutionary properties of the nonarresting cells depend on the measure (or observable) of interest. When examining the average populations sizes in competition simulations, nonarresting and arresting cells display neutral dynamics. The fixation probability of nonarresting mutants, however, is lower than predicted for a neutral scenario, suggesting a selective disadvantage in this setting. For nonarresting cells to gain a selective advantage, additional mechanisms must be invoked in the model, such as small, repeated phases of tissue damage, each resulting in a brief period of regenerative growth. The same properties are observed in a more complex model where it is explicitly assumed that repair and temporary cell cycle arrest are dependent on the cell having sustained DNA damage, the rate of which can be varied. We conclude that repair‐deficient cells are not automatically advantageous in the presence of frequent DNA damage and that mechanisms beyond avoidance of cell cycle delay must be invoked to explain their emergence.

## 1 INTRODUCTION

The tissue environment in vivo is subject to evolutionary processes, in which different cell variants can emerge during the life span of an individual. Some cell variants can predispose the tissue to the development of disease, most notably cancer (Frank & Nowak, 2004). The dynamics and rate of emergence of mutant cells depend on their competitive ability relative to wild‐type cells, because the cellular environment provides limited space and resources, for which cells compete in homeostasis. The growth rate of cells is thought to be an important trait that can influence the Darwinian fitness of cells, with faster growing cells often being considered advantageous (Wodarz & Komarova, 2014). One aspect that determines the growth rate of cells is the speed with which cells progress through the cell cycle. This in turn can depend on the environment and on alterations that are inflicted on the genetic material (Elledge, 1996). If a cell is damaged, cell cycle checkpoints can result in temporary cell cycle arrest, during which the cell has a chance to repair the damage, leading to delayed cell cycle progression and delayed cellular reproduction (Branzei & Foiani, 2008; Hartwell & Weinert, 1989). Mutants can emerge that are characterized by defects in these checkpoint mechanisms (Kastan & Bartek, 2004), which can result in the absence of repair or cell cycle delay. In the presence of frequent cellular damage, it has been hypothesized that such cells could enjoy an increased growth rate, and thus a selective advantage, leading to their emergence. This concept has been termed “don't stop for repairs in a war zone,” according to an analogy in which a “repairing” and a “nonrepairing” car race in an environment in which they are shot upon (Breivik, 2001, 2005; Breivik & Gaudernack, 1999a, 1999b). While the repairing car remains in better condition, it stops for repair too often to be competitive. Although the nonrepairing car accumulates damage, it continues to move, even if slowly, and hence wins the race.

Checkpoint‐deficient cells are characterized by an increased probability to accumulate mutations, which can lead to cellular transformation and initiation of uncontrolled cell growth. They have been termed genetically unstable cells, or “mutator phenotypes” (Boland & Goel, 2005, 2010; Lengauer, Kinzler, & Vogelstein, 1998; Loeb, 1991, 1998, 2001; Loeb & Loeb, 2000). An example of mutator phenotypes is cells that display microsatellite instability (MSI) and that can occur in colorectal tissue and in colorectal cancer (Boland & Goel, 2005, 2010; Thibodeau, Bren, & Schaid, 1993). Microsatellite unstable cells are characterized by the inactivation of mismatch repair (MMR) mechanisms and can thus accumulate point mutations in repeat microsatellite sequences. Inflammatory tissue conditions might be responsible for the emergence of MMR‐deficient cells, an example being the emergence of MMR‐deficient cells in noncancerous colorectal tissue in patients with ulcerative colitis (Brentnall et al., 1996).

While frequent repair of DNA damage and the consequent delay in reproduction have been hypothesized to result in a selective advantage of repair‐deficient cells (Breivik, 2001, 2005; Breivik & Gaudernack, 1999a, 1999b), the effect of cell cycle delay on the evolutionary dynamics of cells has never been rigorously investigated with population dynamic and evolutionary mathematical models, which are central tools in this context. Here, we provide such an investigation. We start by examining the competition dynamics between two cell types: a population that enters temporary cell cycle arrest (referred to as the “arresting population”) and one that does not (referred to as the “nonarresting” cell population). We investigate how delayed reproduction through temporary cell cycle arrest influences the average sizes of the cell populations over time and the probability that a nonarresting cell population invades an arresting population and fixates. This provides first insights into how delayed reproduction can affect the evolutionary dynamics of cells. We subsequently expand this model to explicitly assume that cell cycle arrest is brought about by DNA damage, the intensity of which can be varied.

## 2 EFFECT OF TEMPORARY CELL CYCLE ARREST ON GROWTH RATE AND COMPETITIVE ABILITY OF CELLS

**Figure 1:**
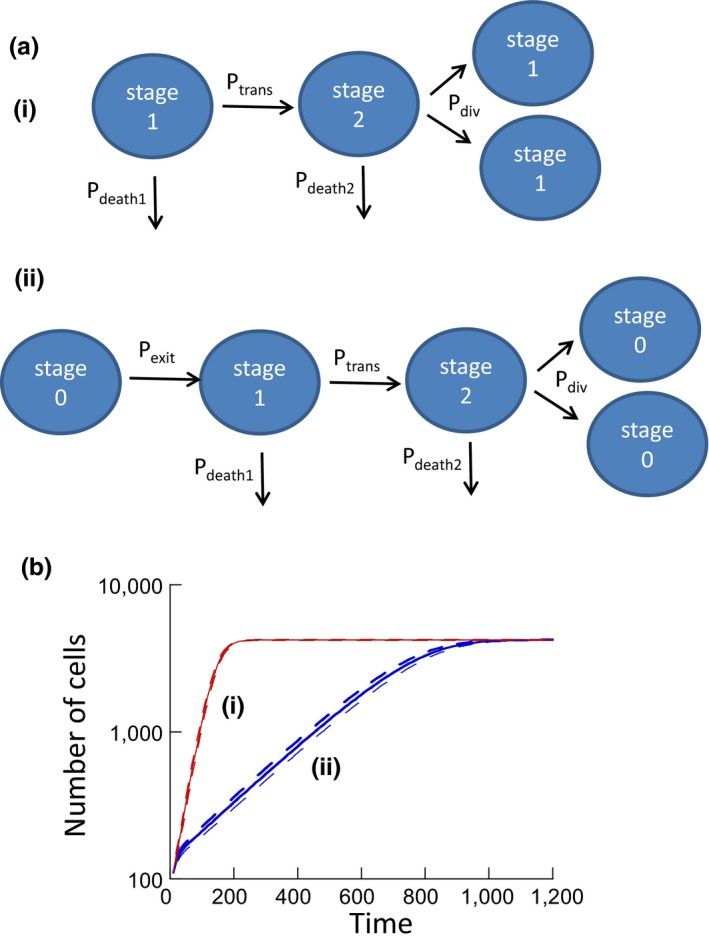
(a) Schematic of the processes in the basic model and the relevant parameters. (i) Processes in the “nonarresting” cell population. (ii) Processes in the “arresting” cell population. See text for details. (b) Simulation of the basic model, showing the growth of an arresting and nonarresting cell population in isolation, that is, not in competition. The simulation was run 5,600 times, and the solid lines show the average trajectories. The dashed lines are the average ± standard deviation. Parameters were chosen as follows. *N* = 4,900; P_div_ = 0.1; P_death1_ = 0.015; P_death2_ = 0.01, P_trans_ = 0.1. For arresting cells, P_exit_ = 0.01

In this section, we use a computational model to investigate the hypothesis that avoidance of temporary cell cycle arrest and DNA repair can be advantageous for the cell. The model contains two populations (Figure 1a): one that temporarily enters cell cycle arrest, and one that does not. For convenience, we will refer to the former as an “arresting cell population” and to the latter as a “nonarresting cell population.” For simplicity, we only distinguish between two basic cell cycle stages (Figure 1a): The second stage is the “dividing stage,” in which cells have a certain probability to divide, and a certain probability to die. Upon division, both cells enter stage 1, which comprises all nondividing stages of the cell cycle. In this phase, cells are characterized by a probability to die and a probability to transition to the dividing stage. These are the processes that apply to the nonarresting cell population. For the arresting cell population, cells enter an arresting “stage 0” upon division. Because these cells are assumed to repair damage, it is assumed that no death occurs and that the cells exit cell cycle arrest with a certain probability to enter stage 1. Hence, in this simplified model, cells either arrest for repair following every cell division, or they never arrest. We do not take into account possible fitness costs that arise from not repairing (due to a higher mutational burden), because we are interested to find out in which way the delay due to temporary arrest alone influences the outcome of competition among cells. These assumptions are implemented as a stochastic agent‐based model, where individual cells are tracked explicitly. The model contains N “spots,” which represents the maximal number of cells that the system can sustain. Each spot can be either empty or contain a cell. At each time step, M individual updates are performed, where M is the total number of cells currently in the system. At each update, a cell is picked at random, and one of the processes described above is selected to occur. The probabilities of the different processes are proportional to their rates, see Figure 1a. When a cell divides, the offspring cell is placed in a randomly chosen spot. If that spot already contains a cell, the division is aborted. This model will be referred to as the “basic model.”

### 2.1 Growth in isolation

The simulation was run and repeated multiple times, and for each time point, the average population sizes were determined and plotted. To characterize the basic growth properties of the two cell strains, we simulated their growth in isolation starting from a relatively low population size, but large enough to render stochastic extinction unlikely (Figure 1b). Exponential growth was initially observed for both populations, followed by convergence to a steady state. As expected, the arresting cell population grew slower than the nonarresting one (Figure 1b).

### 2.2 Competition dynamics

**Figure 2:**
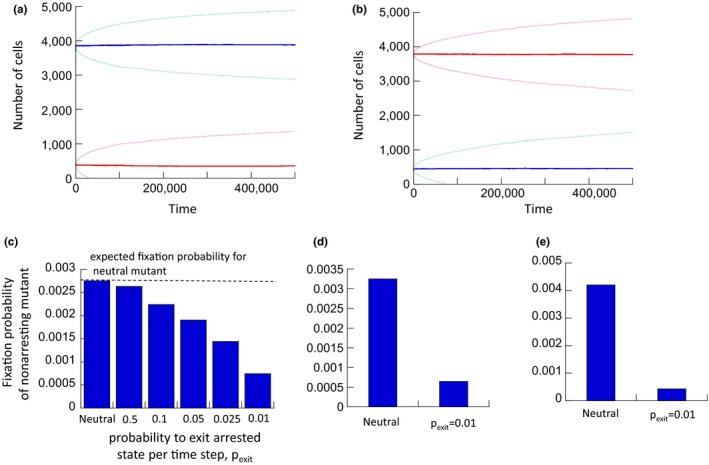
(a) Simulation of the basic model, where the arresting and the nonarresting cell populations compete. The blue and red lines show the average trajectories of arresting and nonarresting cells, respectively, over 5,600 realizations of the simulation. The light curves around the average curves represent average ± standard deviation. Parameters were chosen as follows. *N* = 4,900; P_div_ = 0.1; P_death1_ = 0.015, P_death2_ = 0.01, P_trans_ = 0.1. For arresting cells, P_exit_ = 0.01. (b) Same, but with different initial population abundances. This shows that the equilibrium levels depend on initial population sizes, indicating neutral dynamics. (c) Fixation probability of a single nonarresting cell placed into an established arresting cell population at equilibrium. The simulation was run repeatedly (>10^6^ times), recording the fraction of fixation events. The “neutral” bar is the control simulation where the fixation probability was determined for a mutant that is equivalent to the established cell population (i.e., arresting, with same parameters). The horizontal line indicates the expected fixation probability for a neutral mutant, given by 1/M, where M is the average number of arresting cells in isolation around equilibrium (the average population size over time was determined, because the populations fluctuate stochastically around an equilibrium). The remaining bars show the fixation probability of a nonarresting cell placed into an established arresting cell population characterized by different probabilities to exit the arresting state, P_exit_. The lower the value of P_exit_, the more different the arresting cell population is from the nonarresting one, and the lower the fixation probability. Parameters were as follows: *N* = 900, P_div_ = 0.1; P_death1_ = 0.01, P_death2_ = 0.05, P_trans_ = 0.1 (d, e) Same, but assuming a higher death probability for stage 1 cells, that is, for (d) P_death1_ = 0.015 and for (e) P_death1_ = 0.02. Only the simulation for P_exit_ = 0.01 is compared to the neutral case for simplicity. All differences are statistically highly significant (*p* ≪ .05) using the *z* score for comparing population proportions

Next, we investigated the dynamics when both cell types grew together and competed for space. The simulations were started with different initial abundances of the two cell strains (Figure 2a,b). Contrary to previous notions, we found that the nonarresting cell population was not advantageous, but was competitively neutral with respect to the arresting cell population (Figure 2a,b): The equilibrium levels of the populations were determined by their initial abundances. This suggests that skipping cell cycle arrest and progressing through the cell cycle faster does by itself not confer a selective advantage to the cell population, even though such cells are characterized by a faster exponential growth rate. The reason is that a nonarresting cell on average produces the same number of offspring cells during its life span, regardless of the speed of cell cycle progression.

### 2.3 Fixation probability of nonarresting mutants

Next, we considered a situation where the cell population consisted of arresting cells around their equilibrium population size, into which a single nonarresting “mutant” cell was placed. We investigated the probability with which this mutant became fixated (i.e., comprised 100% of the cell population). This was carried out by repeatedly running the simulation and determining the fraction of runs that resulted in fixation of the mutant, according to the following protocol. The arresting cell population was allowed to equilibrate, and at a defined time point, a single nonarresting cell in stage 1 was introduced into this population. If two populations are neutral, the fixation probability is 1/M, where M is the initial number of cells in the system. This was the case in our simulation if both of the cell populations were identical, that is, if the established and the “mutant” cell populations were both arresting, with identical parameters (the bar marked “neutral” in Figure 2c). Results become different, however, if the established cell population is arresting, while the mutant cell population is nonarresting. Now, the numerically obtained fixation probability is lower than 1/M, that is, the nonarresting cell population behaves like a disadvantageous mutant (Figure 2c). These simulations were run assuming different probabilities with which the established cells exit the arrested state (different values of *p*_exit_). The faster the cells exit the arrested stage (higher *p*_exit_), the shorter this phase lasts, and the closer the properties of the arresting cell population become to those of the nonarresting cell population. The longer the duration of cell cycle arrest (the lower the value of *p*_exit_), the larger is the difference between the two cell populations, and the lower the fixation probability of the nonarresting cells, compared to the neutral case. Therefore, in the context of fixation probabilities, nonarresting cells invading an arresting cell population act like disadvantageous mutants, with the extent of the disadvantage becoming more pronounced for longer cell cycle arrest durations of the established population. Figure 2d,e repeats this analysis assuming higher death rates for cells in stage 1 (only considering *p*_exit_ = .01 for simplicity). This shows that the effect becomes more pronounced for larger stage 1 death rates (as shown mathematically in the Supporting Information).

## 3 ANALYTICAL INSIGHTS INTO FIXATION PROBABILITY

The numerical observation that nonarresting cells act like disadvantageous mutants when invading an arresting cell population from low numbers was a surprising result. To obtain more insights, we performed mathematical analysis in the context of three different stochastic modeling approaches: the Moran process (both the death–birth and the birth–death formulations) and the contact process (see Supporting Information for full details of the analysis).

The Moran process model is a very useful theoretical tool, because it is analytically more tractable than the basic model considered above. It assumes a constant population of cells and can be formulated in the following two ways. (i) *The death–birth process*: At each update, a cell is chosen at random with the probability proportional to its death rate and is immediately replaced by the progeny of another cell that is chosen for reproduction based on its division rate, (ii) *The birth–death process*: At each update, first a cell is chosen for reproduction (again, with the probability proportional to its reproduction rate) and then a cell is chosen for death (with the probability proportional to its death rate), to be replaced by the progeny of the cell that had reproduced. Despite very similar formulations, the two update rules may lead to noticeably different results, see, for example, Kaveh, Komarova, and Kohandel (2015).

Let us examine the behavior of competition dynamics of two types of cells: The wild‐type cells enter cell cycle arrest immediately upon division and exit this state with a given rate, whereas mutants never enter cell cycle arrest. Intuitively, the difference between the two populations can be described as a difference in cellular turnover: The wild types turn over slower than the mutants; that is, they divide and die with a certain delay. This motivated us to start theoretical analysis by considering a simple model where no cell cycle arrest is incorporated, but instead mutants have birth and death rates that are α times faster than those of the wild‐type cells (with constant α > 1). It turned out in both formulations of the Moran process, and mutant cells experience negative selection and fixate with a probability smaller than 1/*N*. The reason for this is subtle and boils down to small changes in the competition dynamics that follow individual division and death events. Superficially, one could expect mutants to be neutral because although they die more often than wild‐type cells, they also divide more often, and the two effects should cancel each other. It turns out, however, that there is a certain imbalance of probabilities. In the death–birth process, consider the probabilities for the mutant population to increase and to decrease by one cell:

1. An increase happens if a wild‐type cell dies and a mutant divides, and the death of a wild‐type cell slightly increases the odds for a mutant cell to be chosen for division.
2. A decrease in the mutant population is observed if a mutant death is followed by a wild‐type cell division, and the mutant death increases the odds for a wild‐type cell to be chosen for division.

It is easy to see that elimination of a *stronger* competitor (as in the latter sequence of events) changes the probabilities more, which means that a decrease in a mutant population becomes more likely than an increase, thus making mutants disadvantageous. A similar argument can also be carried out for the birth–death process, resulting in mutants being selected against.

In a more realistic *contact process*, the population is not rigidly held constant, and different events simply happen at their rates. In reality, however, the cell colony fluctuates around an equilibrium level, and birth and death events balance each other on average. As both birth followed by death and death followed by birth result in a negative mutant selection, it is not surprising that in the contact process, the probability of fast‐turning mutants to fixate is smaller than 1/*N*.

Intuition developed by considering the competition dynamics of fast and slow cells is helpful when we return to studying the actual process where mutants avoid cell cycle arrest. An argument similar to the one presented above can be developed for the two formulations of the Moran process. Interestingly, the death–birth process in this case has no selection for or against nonarresting cells, which fixate with probability exactly equal to 1/*N*. The birth–death process, however, works out to exhibit selection against the mutants. In the more natural contact process, different pairs of events occur, and the net effect is negative, resulting in the nonarresting mutant fixating with a probability lower than 1/*N*. Details of the calculations, as well as parameter dependence of the trends, can be found in the Supporting Information.

## 4 SELECTION OF NONARRESTING CELLS BY DISTURBANCE OF HOMEOSTASIS

The above results indicate that skipping cell cycle arrest/repair is by itself not advantageous for cells. In fact, it is disadvantageous in the context of mutant fixation. This brings up the question about possible microenvironmental conditions under which nonarresting cells become advantageous, because this would significantly promote the emergence of mutator phenotypes. To investigate this, we go back to our basic model discussed in Section 2. It turns out that in the presence of repeated tissue disturbance, nonarresting cells can in fact act like advantageous types. This is explored as follows.

### 4.1 Competition dynamics

**Figure 3:**
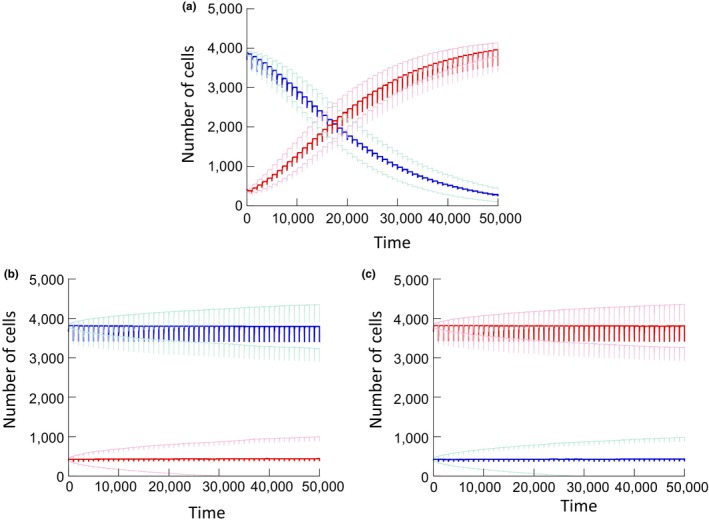
Simulation of the basic model, assuming repeated disturbance of tissue homeostasis. Every 1,000 time steps, the overall population was reduced by 10%, leading to subsequent tissue regeneration (seen in the rugged shape of the lines). (a) Competition dynamics of nonarresting and arresting cell populations. The blue and red lines show the average trajectories of arresting and nonarresting cells, respectively. 5,600 realizations of the simulation were run. The light curves around the average curves represent average ± standard deviation. Parameters were chosen as follows. *N* = 4,900; P_div_ = 0.1; P_death1_ = 0.015; P_death2_ = 0.01, P_trans_ = 0.1. For arresting cells, P_exit_ = 0.01. (b) Same simulation, but with two identical arresting populations. (c) Same as (b), but with different initial population sizes, indicating neutral dynamics

When we investigated the average dynamics of the arresting and nonarresting cells with the basic model in Section 2, we found the two cell populations to be neutral with respect to each other, as described before (Figure 2a,b). Here, we repeated these simulations assuming that the overall number of cells was reduced by a certain percentage (e.g., 10%) at regular time intervals (Figure 3a). When the cell population is reduced, renewed growth can occur to fill the space. Because nonarresting cells grow faster than arresting cells during this phase, their relative abundance is elevated to a certain degree following each disturbance event. With each tissue disturbance, the relative abundance of the nonarresting cells progressively increases until they become the dominant population (Figure 3a). To ensure that this result is indeed due to the difference in the ability of cells to enter temporary cell cycle arrest, and not simply due to the tissue disturbance alone, we repeated the simulations assuming that the two cell strains are truly competitively neutral, that is, both populations consist of arresting cells with identical parameters (Figure 3b,c). In this case, no selection of nonarresting cells occurs. Instead, the equilibrium depends on the initial population sizes, thus indicating neutral dynamics.

### 4.2 Fixation probability of nonarresting mutants

**Figure 4:**
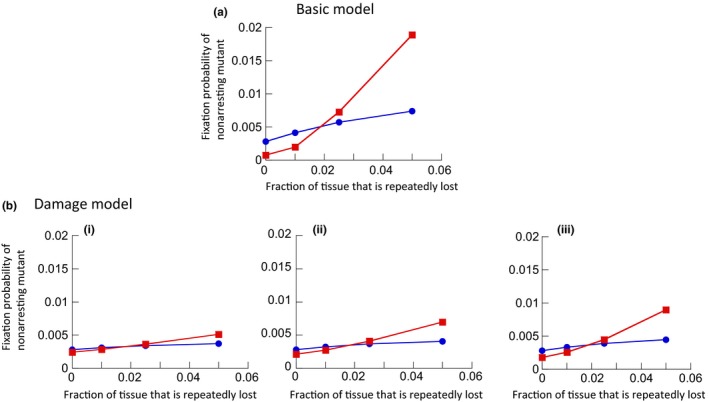
(a) Basic model: Fixation probability of one nonarresting mutant placed into a population of arresting cells at equilibrium (red line), running >10^6^ iterations of the simulation. The fixation probability is plotted against the amount of tissue homeostasis disturbance, expressed as the fraction of cells that is removed every 300 time steps. The blue line indicates the neutral control, where the fixation probability of one arresting cell was determined when placed into an established arresting population with identical parameters. Parameters were chosen as follows. *N* = 900, P_div_ = 0.1; P_death1_ = 0.01, P_death2_ = 0.05, P_trans_ = 0.1, P_exit_ = 0.01. (b) Same but for the damage model, assuming different genetic hit rates: (i) *u* = 0.1, (ii) *u* = 0.2, (iii) *u* = 0.3. The differences between the non‐neutral and neutral simulations were statistically highly significant (*p* ≪ .05) using the *z* score for comparing population proportions

Assuming different severity levels of repeated tissue disturbance, we investigated the fixation probability of a single nonarresting mutant, placed into a population of arresting cells at equilibrium (Figure 4a). It was assumed that the arresting cells exited the arrested state with a probability *p*_exit_ = .01. The red line depicts the fixation probability of the nonarresting mutant, while the blue line depicts the neutral control scenario; that is, the fixation probability for a truly neutral mutant that was assumed to also arrest, with parameters identical to the established cell population. In the absence of tissue damage, we again observe that the fixation probability of the nonarresting cell population is lower than for a neutral mutant (Figure 4a). When the level of tissue disturbance, however, crossed a threshold, the fixation probability for the nonarresting cell population became larger than that observed for a neutral mutant (Figure 4a). In other words, with sufficient tissue disturbance, the nonarresting cell population acted like an advantageous mutant. Note that more pronounced tissue disturbance increases the fixation probability even for the neutral scenario, because the population size of the resident population is temporarily reduced. The fixation probability of the nonarresting mutant, however, increases to a larger extent due to the faster growth rate of the nonarresting mutant during periods of repopulation.

## 5 MODEL WITH DNA DAMAGE

The above model investigated the competition and evolutionary dynamics between an arresting and a nonarresting cell population. This was a useful approach to learn about the effect of temporary cell cycle arrest on the competitive ability of cells. In biological terms, this can be thought of as corresponding to a scenario where upon every cell division, a cell needs to enter cell cycle arrest to repair some mistake. In reality, however, this should be modeled in a more complex way such that cell cycle arrest and repair is only induced with a certain probability that is determined by the rate with which cells become damaged. Here, we modify the basic model to include this added complexity. Thus, upon cell division in stage 2, cells belonging to the “arresting population” have a probability p_hit_ to receive damage and to enter stage 0 (temporary cell cycle arrest). Otherwise, these cells enter stage 1 and do not arrest. As before, cells belonging to the nonarresting population never enter temporary cell cycle arrest. This model will be referred to as the “damage model.”

We find that the results reported in the context of the basic model remain largely robust, with the extent of the effect dependent on the probability of arresting cells to receive damage (*p*_hit_). Figure 4b shows the fixation probability for a nonarresting mutant cell population (red) compared to the fixation probability of a neutral mutant (blue), derived from the damage model. This was determined in the absence of tissue disturbance, and in the presence of repeated tissue disturbance, as before. The trends are identical to those observed with the basic model (Figure 4a), although less pronounced. In the absence of repeated tissue disturbance, the nonarresting cell population acts like a disadvantageous mutant, with the fixation probability lower than observed for neutral mutants. If the extent of repeated tissue disturbance crosses a threshold, the situation is reversed and the nonarresting cells act like advantageous mutants. The trend becomes more pronounced going from panel (i) to panel (iii) in Figure 4b, because an increasing probability to receive damage is assumed in successive panels (increase in the value of *p*_hit_).

**Figure 5:**
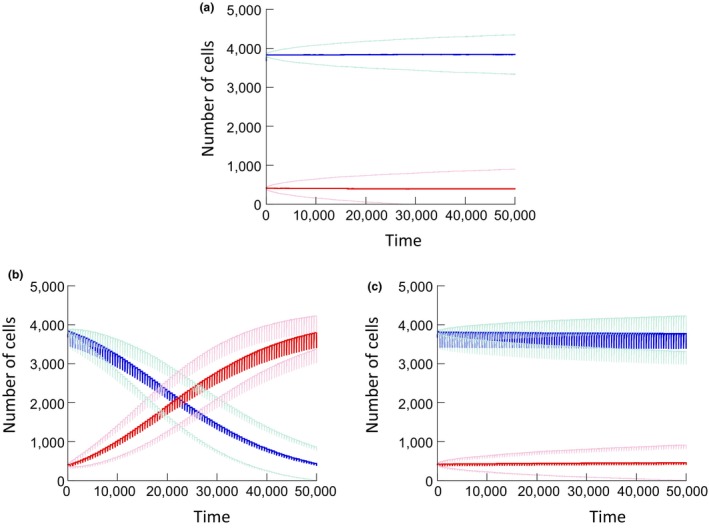
(a) Damage model: Simulation of the average population trajectories, over 5,600 realizations of the simulation. The blue and red lines show the average trajectories of arresting and nonarresting cells, respectively. The light curves around the average curves represent average ± standard deviation. Parameters were chosen as follows. *N* = 4,900; P_div_ = 0.1; P_death1_ = 0.015, P_death2_ = 0.01, P_trans_ = 0.1, *u* = 0.1. For arresting cells, P_exit_ = 0.01. (b) Same simulation, but assuming a reduction of the overall cell population by 10% every 500 time steps (seen as rugged lines). Color coding is the same as before. (c) Same, but assuming a neutral scenario where both populations are arresting with identical parameters

The properties of the average growth dynamics of arresting and nonarresting cell populations competing with each other are also qualitatively similar to the results shown for the basic model. In the absence of repeated tissue disturbance, neutral dynamics are observed (Figure 5a). In the presence of repeated tissue disturbance, nonarresting cells gain in abundance relative to the arresting population over time (Figure 5b). This is in contrast with Figure 5c that shows neutral dynamics of two identical, arresting populations in the presence of tissue disturbance.

## 6 DISCUSSION AND CONCLUSION

Checkpoint‐deficient cells that avoid repair and associated cell cycle delays have been hypothesized to enjoy a selective advantage in settings where the checkpoint is frequently induced, because this allows them to divide with a faster rate compared to cells with intact checkpoints that trigger repair and cell cycle delay. This was also called the “don't stop for repairs in a war zone hypothesis” (Breivik, 2001, 2005; Breivik & Gaudernack, 1999a, 1999b). Using computational models, we showed that this mechanism might not work. In the contrary, a mutant cell that fails to slow down cell cycle progression in the face of DNA damage is predicted to behave either like a neutral or a disadvantageous mutant, depending on the evolutionary measure considered.

An aspect not included in our model is the potential fitness cost of nonrepairing cells due to accumulation of deleterious mutation. This was carried out deliberately to provide the largest possible advantage to the nonrepairing cells. Even under these favorable assumptions, our models showed that nonrepairing cells were not advantageous in standard conditions. Adding fitness cost due to mutant accumulation in nonrepairing cells only reduces the potential of such cells to evolve and thus strengthens our results.

In the light of these arguments, it is important to investigate the forces that select for the emergence of repair‐deficient cells, because the emergence of such cells occurs in precancerous conditions such as ulcerative colitis (Brentnall et al., 1996) and of course in tumors. Our work offers one possible explanation: if tissue homeostasis is repeatedly disturbed (e.g., by microenvironmental conditions), tissue space is freed up, allowing populations to expand. Because this expansion occurs faster for nonarresting cells (Figure 1b), such cells would gradually increase relative to repairing (and hence arresting) populations. This could lead to the gradual selection of repair‐deficient cells. An alternative mechanism could be that a repair checkpoint can also induce apoptosis. For example, the mismatch repair pathway can lead to either repair with temporary cell cycle arrest, or it can lead to apoptosis, depending on the nature and extent of the DNA damage (Wu, Gu, Wang, Geacintov, & Li, 1999). MMR‐deficient cells would then not only avoid repair, but also avoid programmed cell death in response to DNA damage. In our modeling framework, any reduction in the rate of cell death would lead to a strong selective advantage (not shown here). Therefore, repair deficiency could hitchhike on the selective advantage obtained by cells through escape from apoptosis. This is likely what accounted for the selection of MMR‐deficient colorectal cancer cells during in vitro experiments when exposed to carcinogens (Bardelli et al., 2001; Hickman & Samson, 1999; O'Brien & Brown, 2006). Thus, treatment of cells in vitro with the methylating agent N‐methyl‐N’‐nitro‐N‐nitrosoguanidine (MNNG) resulted in the emergence of MMR‐deficient cells that were resistant to MNNG (Bardelli et al., 2001). In these experiments, however, cells in the culture were subjected to intense selection pressure because essentially all MMR‐proficient cells died in the culture, leaving behind only MMR‐deficient cells. Such conditions are unlikely to apply in vivo, where cells are exposed to much lower doses of carcinogens. It is unclear how exactly a decision is made by the cell about whether to repair a given damage or whether to trigger apoptosis (Harfe & Jinks‐Robertson, 2000). At lower levels of damage, the cell is probably more likely to repair the damage than to undergo cell death (Branzei & Foiani, 2008; Nowsheen & Yang, 2012).

It is also interesting to consider our results in the context of checkpoint robustness against escape. It is clear from the above discussion that a checkpoint that induces cell death due to damage (or permanent cell cycle arrest, which is functionally the same) is prone to the evolution of escape. If an escape mutant has been created, it has a selective advantage and a high likelihood to invade. This, however, does not apply to escape from checkpoint‐induced repair and temporary cell cycle delay, as demonstrated in this article. Such mutants do not enjoy a selective advantage according to our models, meaning that it will take longer for them to emerge. Hence, from an escape point of view, repair should be the preferred defense against genetic alterations because evolution of escape is expected to take longer. Apoptosis or permanent cell cycle arrest should be reserved for damage that cannot be successfully repaired.

Finally, we would like to discuss implications of our work for aspirin‐induced chemoprevention of colorectal cancers (Chan et al., 2008, 2012; Cuzick et al., 2009), especially in patients with Lynch syndrome (Burn, Mathers, & Bishop, 2013; Burn et al., 2011). An important effect of aspirin is that it reduces the degree of inflammation in tissues. Inflammation has been considered a central component of carcinogenesis, and inflammation has been shown to promote the occurrence of MMR deficiency in various ways (Colotta, Allavena, Sica, Garlanda, & Mantovani, 2009). Another impact of inflammation could be a general and ongoing disturbance of healthy tissue homeostasis, due to toxicity to cells (Coussens & Werb, 2002). As suggested by our models, the presence of ongoing homeostasis disturbance can lead to a temporary growth advantage for repair‐deficient cells, leading to their gradual selection over time. This could be a potential mechanism that selects for MMR‐deficient cells in inflamed, but otherwise healthy tissue (Brentnall et al., 1996). By reducing inflammation, aspirin could reduce this ongoing disturbance of homeostasis and thus delay the evolution of repair‐deficient cells. In other words, aspirin might alter the tissue environment such that repair‐deficient cells are disadvantageous.

## DATA ARCHIVING STATEMENT

The article is based on computational modeling, with all information provided in the article.
