# Supplementary material for: Effect of cell cycle duration on somatic evolutionary dynamics
Source: Evol Appl. 2017 Oct 12;10(10):1121–9. doi: 10.1111/eva.12518 (PMC5680637; doi:10.1111/eva.12518)
Supplement: Supplementary file 1 [file EVA-10-1121-s001.pdf]

Effect of cell cycle duration on somatic evolutionary dynamics

Dominik Wodarz, Ajay Goel, and Natalia L. Komarova

Contents

1 The role of cell turnover in mutant fixation **1**
- 1.1 Moran process 1
- 1.2 Intuitive explanation 2
- 1.3 Contact process and turnover differences 4

2 The absence of arresting state leads to selective disadvantage **5**
- 2.1 The contact model with stage zero 6
- 2.2 The connection between cell turnover and the arresting state 7
- 2.3 The Moran process with an arresting state 9
- 2.4 The limit of $\nu \to 0$ 11

1 The role of cell turnover in mutant fixation

1.1 Moran process

Consider competition dynamics of two cellular populations. The wild type population is characterized by division rate r_x and death rate d_x, and the mutant population has equivalent parameters r_y and d_y. We will focus of the question of mutant fixation, specifically as a function of the relative turnover of the two populations. If

$$r_y = \alpha r_x, \quad d_y = \alpha d_x, \tag{1}$$

then mutants have a higher turnover compared to the wild type if $\alpha > 1$, and they have a lower turnover if $\alpha < 1$. The kinetic parameters of the two types are identical if $\alpha = 1$.

We will start by formulating the Moran process of cellular dynamics. At each update, one death event and one birth event happen, to preserve the total population at a constant size, N. There are two different implementation of the Moran model, death-birth and birth-death process, see [1]. We will present the death-birth version in detail and then show that similar patterns are observed in the birth-death model.

The state of the system is characterized by the number of mutants, j (such that the number of wild type cells is given by $i = N - j$). The transition probabilities are:

$$P_{j\to j+1} = \frac{d_x i}{d_x i + d_y j}\,\frac{r_y j}{r_x(i-1) + r_y j}, \tag{2}$$

$$P_{j\to j-1} = \frac{d_y j}{d_x i + d_y j}\,\frac{r_x i}{r_x i + r_y (j-1)}, \tag{3}$$

$$P_{j\to j} = 1 - P_{j\to j+1} - P_{j\to j-1}, \tag{4}$$

with the rest of transitions having zero probability. In equation (2), the first multiplier in the right hand side is the probability of a wild type cell to die, and the second multiplier is the probability for a mutant to divide; note that because a division happens after a death, the number of wild type cells available for divisions is decreased by one. Similarly, in (3), the first multiplier in the right hand side is the probability of a mutant to die, and the second multiplier is the probability for a wild type to divide (where the number of mutants is decreased by one due to the preceding death event). Denoting by ρ_j the probability for mutants to fixate starting from j cells, we have

$$\rho_j = P_{j\to j+1}\rho_{j+1} + P_{j\to j-1}\rho_{j-1} + P_{j\to j}\rho_j, \quad 0 < j < N, \tag{5}$$

with the obvious boundary conditions

$$\rho_0 = 0, \quad \rho_N = 1.$$

It is convenient to rewrite equation (5) as

$$\rho_j(P_{j\to j+1} + P_{j\to j-1}) = P_{j\to j+1}\rho_{j+1} + P_{j\to j-1}\rho_{j-1}, \quad 0 < j < N.$$

Solving this system gives us the probability of mutant fixation starting from one cell, ρ_1. Under condition (1) we obtain the simple result,

$$\rho_1 = \frac{1}{N}\frac{2}{1+\alpha}.$$

We observe immediately that if the mutants are characterized by a faster turnover compared to wild type cells, their probability of fixation starting from one cell is smaller than $1/N$. Conversely, if the mutants turn over slower, they fixate with a probability larger than $1/N$.

In general, we have

$$\rho_j = \frac{j}{N}Q_{j,\alpha}, \quad Q_{j,\alpha} = \frac{N - \frac{1+\alpha}{2} - j\frac{1-\alpha}{2}}{(N-1)\frac{1+\alpha}{2}}.$$

One can see that because $Q_{j,1} = 1$ and $\frac{dQ_{j,\alpha}}{d\alpha} = -\frac{2(N-j)}{(1+\alpha)^2(N-1)} < 0$, the probability of mutant fixation is greater than j/N if $\alpha < 1$ and it is smaller than j/N if $\alpha > 1$. So we conclude that the mutant characterized by a faster (slower) turnover compared to the wild type will fixate with a probability lower (higher) than its initial frequency, and thus behaves as if it is selected against (selected for).

1.2 Intuitive explanation

The reason for this behavior can be seen by evaluating the quantity

$$\frac{P^{db}_{j\to j+1}}{P^{db}_{j\to j-1}} = \left(\frac{d_x r_y}{d_y r_x}\right)\left(\frac{r_x i + r_y(j-1)}{r_x(i-1) + r_y j}\right),$$

see equations (2-3). The first multiplier on the right is 1 under condition (1). The second multiplier is equal to 1 if $\alpha = 1$. This means that if mutants have the same kinetic parameters

as the wild type cells, their dynamics is pure drift, with probability to increase balanced exactly by the probability to decrease. If however $\alpha \neq 1$, a selection-type force is observed. If $\alpha > 1$ (and $r_y > r_x$), the expression above is smaller than unity, and

$$P^{db}_{j\to j+1} < P^{db}_{j\to j-1},$$

that is, mutants are more likely to decrease than to increase. In the opposite case where $\alpha < 1$, mutants are more likely to increase.

The reason for this pattern can be seen in the following way. Let us assume that the mutants have a faster turnover ($\alpha > 1$), and suppose that in some hypothetical process (denoted below by star), in the equivalent of formulas (2-3), the probability of the 2nd event (divisions) in unaffected by the first event (death):

$$P^*_{j\to j+1} = \frac{d_x i}{d_x i + d_y j}\,\frac{r_y j}{r_x i + r_y j} < P^{db}_{j\to j+1} = \frac{d_x i}{d_x i + d_y j}\,\frac{r_y j}{r_x (i-1) + r_y j}, \quad (6)$$

$$P^*_{j\to j-1} = \frac{d_y j}{d_x i + d_y j}\,\frac{r_x i}{r_x i + r_y j} < P^{db}_{j\to j-1} = \frac{d_y j}{d_x i + d_y j}\,\frac{r_x i}{r_x i + r_y (j-1)}. \quad (7)$$

In this case, under condition (1), we have $P^*_{j\to j+1} = P^*_{j\to j-1}$. This is because the mutants have a higher chance (per cell) to be chosen for death, and they also have a higher chance to be chosen for division, and the two trends exactly cancel each other.

In reality, the changes in the division probabilities induced by the first of the events (a death) break this symmetry. As shown in (6), a result of a wild type cell dying, the probability of a mutant to divide will increase (because the mutant now has lost one of its competitors). Similarly, as shown in (7), the probability of a wild type division will increase as a consequence of the previous death event, because the wild type cells will also lose one of their competitor. The key is that the increase in $P^{db}_{j\to j-1}$ is larger than the increase in $P^{db}_{j\to j+1}$, because in the case of inequality (7), wild types lose a *stronger* competitor (a mutant, which has a larger division rate, $r_y > r_x$). The increase in $P^{db}_{j\to j+1}$ is relatively smaller because in the case of inequality (6), mutants lose a *weaker* competitor (a wild type cell, which has a smaller division rate).

A similar argument can be developed for the birth-death process. There, the first event (division) decreases the probability of the second event (death):

$$P^{bd}_{j\to j+1} = \frac{r_y j}{r_x i + r_y j}\,\frac{d_x i}{d_x i + d_y (j+1)}, \quad (8)$$

$$P^{bd}_{j\to j-1} = \frac{r_x i}{r_x i + r_y j}\,\frac{d_y j}{d_x (i+1) + d_y j}, \quad (9)$$

but the decrease in $P^{bd}_{j\to j+1}$ is larger than the decrease in $P^{bd}_{j\to j-1}$ if the mutants turn over faster. In other words, if $\alpha > 1$,

$$P^{bd}_{j\to j+1} < P^{bd}_{j\to j-1}.$$

This again indicates the presence of negative selection pressure.

1.3 Contact process and turnover differences

Next, we formulate the contact process where the mutants differ from the wild type cells by their turnover rate according to (1). Denote the number of wild type cells as i and the number of mutant cells as j, we define the total event rate:

$$\mathcal{N} = \begin{cases} (r_x W + d_x)i + (r_y + d_y)j, & 0 \le i + j + k < N, \\ d_x i + d_y j, & i + j + k \ge N, \end{cases}$$

where multiplier W represents cellular competition. The usual logistic model has

$$W(i, j, k) = 1 - \frac{i + j}{K},$$

where $K < N$ is the carrying capacity; divisions gradually become less likely as the population approaches K. Alternatively, we could simply use

$$W(i, j) = \begin{cases} 1, & 0 \le i + j < N, \\ 0, & i + j \ge N. \end{cases} \tag{10}$$

In this case the divisions proceed at a constant per cell rate until the population size N is reached, at which point the divisions stop. Both models are characterized by similar properties when it comes to mutant fixation. The following processes take place:

$$\begin{aligned} P_{(i,j)\to(i-1,j)} &= d_x i/\mathcal{N}, \\ P_{(i,j)\to(i,j-1)} &= d_y j/\mathcal{N}. \end{aligned} \tag{11}$$

In addition, if $0 < i + j + k < N$, we also have

$$\begin{aligned} P_{(i,j)\to(i+1,j)} &= r_x W i/\mathcal{N}, \\ P_{(i,j)\to(i,j+1)} &= r_y W j/\mathcal{N}. \end{aligned}$$

In the process described here all the relevant (and reachable) states (i, j) are inside the simplex S with $i + j \le N$ and $i, j \ge 0$. We can calculate the probability of mutant fixation starting from state (i, j), $\rho_{(i,j)}$, from system

$$\rho_{(i,j)} = \sum_{(i',j')\in S} P_{(i,j)\to(i',j')}\ \rho_{(i',j')}, \quad (i, j) \in S, \quad j \ne 0, \quad i \ne 0, \tag{12}$$

with the additional set of conditions:

$$\rho_{(i,j)} = \begin{cases} 1, & i = 0, \quad j \ne 0, \\ 0, & j = 0, \quad i \ne 0. \end{cases}$$

The latter equation states the definition of mutant fixation and extinction: mutant fixation is reached when the only nonzero population is mutant, and mutant extinction occurs when $j = 0$ but the total population is nonzero. State $(0, 0)$ is is principle reached from either a mutant extinction state of a mutant fixation state, but does not enter system (12).

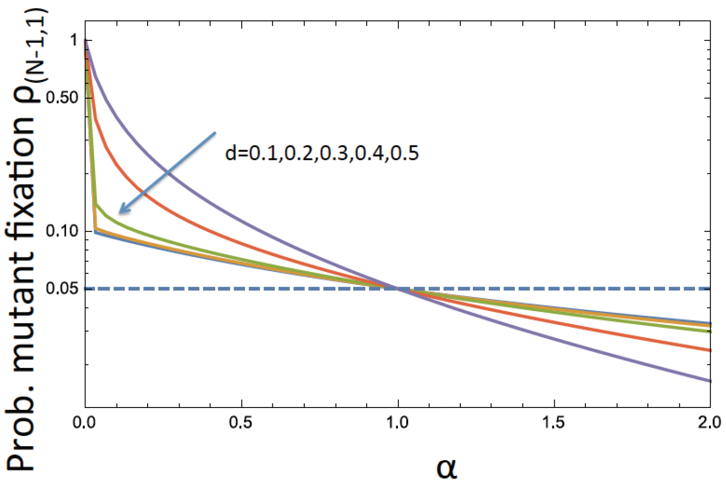

Figure 1: Contact process with different turnover rate of mutants. Mutant fixation probability, $\rho_{N-1,1}$, as a function of the turnover parameter, α, equation (1), for several values of the death rate d. Dashed horizontal lines show the neutral fixation probability, $1/N$. The other parameters are $N = 20, r = 1, W = 1$.

We have set the mutants and the wild type cells to have the kinetic parameters as defined in (1), and solved system (12) for fixation probabilities. The results turn out to be qualitatively very similarto this obtained for the Moran process. Figure 1 illustrates the findings in the case of $N = 20$, by plotting the mutant fixation probability, $\rho_{N-1,1}$ as a function of the turnover parameter, α, equation (1), for several values of the death rate d. We can see that fixation probability is lower than that for a neutral mutant if the mutants have a faster turnover compared to the wild type cells, and the probability of fixation is greater than $1/N$ for slower mutants. This effect is the strongest for larger death rates, d.

The explanation of these results is similar to the explanation offered by the Moran model. In the contact process, although we do not have a rigid population size conservation where each death is necessarily followed by a division, on average, in a contact process near the equilibrium, divisions and death events alternate, which leads to similar dynamics. Since both pairs (division-death and death-divisions) are predicted to favor the downward movement of the number of mutants, the contact process shows the same tendency.

2 The absence of arresting state leads to selective disadvantage

So far we have shown that mutants that are characterized by a faster turnover compared to the wild type cells, experience a selective disadvantage. In the actual system studied here there is no explicit difference between division and death rates of wild type and mutant cells. Instead, a difference in the life history is studied. Let us assume that both wild types and mutants divide at rate r and die at rate d, but upon division, wild types enter a temporary arresting state (stage zero). In this state, a cell does not divide or die, but it can exit this state at a rate ν. We assume that only wild type cells enter stage zero; mutants do not enter

that state.

2.1 The contact model with stage zero

We begin by formulating the contact process of competition between wild types that enter stage zero and mutant cells that do not. Given that the number of active wild type cells is i, the number of wild type cells in stage zero is k, and the number of mutant cells is j, we define the total event rate:

$$\mathcal{N} = \begin{cases} (r_x W + d_x)i + (r_y + d_y)j + \nu k, & 0 \leq i+j+k < N, \\ d_x i + d_y j + \nu k, & i+j+k \geq N. \end{cases}$$

where multiplier W represents cellular competition. As indicated before, two particular models are considered: the usual logistic model that has

$$W(i,j,k) = 1 - \frac{i+j+k}{K},$$

and the model where

$$W(i,j,k) = \begin{cases} 1, & 0 \leq i+j+k < N, \\ 0, & i+j+k \geq N. \end{cases} \tag{13}$$

The following processes take place:

$$\begin{aligned} P_{(i,j,k)\to(i-1,j,k)} &= d_x i/\mathcal{N}, \\ P_{(i,j,k)\to(i,j-1,k)} &= d_y j/\mathcal{N}, \\ P_{(i,j,k)\to(i+1,j,k-1)} &= \nu k/\mathcal{N}. \end{aligned}$$

In addition, if $0 < i+j+k < N$, we also have

$$\begin{aligned} P_{(i,j,k)\to(i-1,j,k+2)} &= r_x W i/\mathcal{N}, \\ P_{(i,j,k)\to(i,j+1,k)} &= r_y W j/\mathcal{N}. \end{aligned}$$

In the process described here all the relevant (and reachable) states (i,j,k) are inside the simplex S with $i+j+k \leq N$ and $i,j,k \geq 0$. We can calculate the probability of mutant fixation starting from state (i,j,k), $\rho_{(i,j,k)}$, from system

$$\rho_{(i,j,k)} = \sum_{(i',j',k')\in S} P_{(i,j,k)\to(i',j',k')}\ \rho_{(i',j',k')}, \quad (i,j,k) \in S, \quad j \neq 0, \quad i+k \neq 0, \tag{14}$$

with the additional set of conditions:

$$\rho_{(i,j,k)} = \begin{cases} 1, & i+k=0, \quad j \neq 0, \\ 0, & j=0, \quad i+k \neq 0. \end{cases}$$

We have set the mutants and the wild type cells to have the same kinetic parameters: $d_x = d_y = d$, $r_x = r_y = r$, and solved system (22) for fixation probabilities. It turns out that starting from one mutant cell, the mutants fixate with probability lower than $1/N$. Figure 2 illustrates the findings in the case of $N = 40$, by plotting the mutant fixation probability,

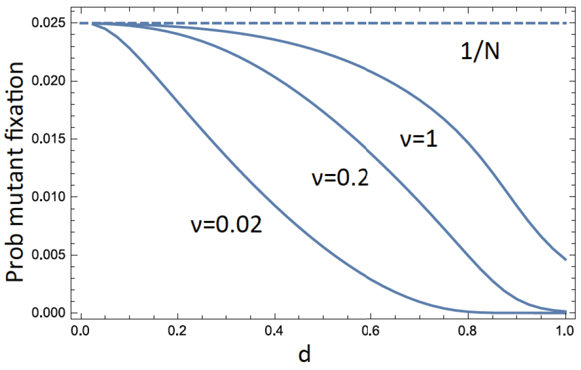

Figure 2: Mutant fixation probability, $\rho_{N-1,1,0}$, as a function of death rate, d, for several values of ν (the rate of transition back from stage zero). $N = 40, r = 1, W = 1$. Dashed horizontal line shows the neutral fixation probability, $1/N$.

$\rho_{N-1,1,0}$ as a function of death rate, d, for several values of ν (the rate of transition back from stage zero). We can see that fixation probability is lower than that for a neutral mutant, indicating that mutants that avoid the arresting state are selected against. The strength of this effect changes with parameters. In particular, it is the strongest for death rates, d, comparable with the division rate, $r = 1$. It disappears in the limit where $d \to 0$, such that $\lim_{d\to 0} \rho_{N-1,1,0} = 1/N$. This is intuitively clear. As the deaths become very rare, the system has time to adjust to fill up all the spots on the grid, and all the newly divided wild type cells come out of stage zero. This way, when the next event (death) happens, the population consists only of mutants and wild type cells, none in stage zero, and all with exactly the same kinetic parameters, thus ensuring that mutants are neutral.

Another important limit is $\nu \to 0$. In this case, the limiting value for $\rho_{i,j,k} = 0$ for $k > 0$ (not shown in the figure). In other words, if there are any cells in arrest initially in the system, mutants cannot fixate. In the absence of stage zero cells at the initial stage, mutant fixation is possible but unlikely; this limit is investigated further below.

The closer the death rate is to the reproduction rate, and also the smaller the rate at which cells in stage zero "wake up" (ν), the stronger the negative effect of selection on the mutants. In order to explain this effect, we need to draw a parallel between this system and the one considered in Section 1.

2.2 The connection between cell turnover and the arresting state

Let us consider the contact process in the absence of mutants and determine the steady state. This can be done by setting the expected change in the population sizes at the steady state to zero:

$$\Delta i = -di - riW + \nu k = 0, \tag{15}$$
$$\Delta k = 2riW - \nu k = 0. \tag{16}$$

Using the logistic expression for $W = 1 - (i+k)/K$, we obtain the solution

$$\bar{i} = K\left(1 - \frac{d}{r}\right)\frac{\nu}{2d+\nu}, \quad \bar{k} = K\left(1 - \frac{d}{r}\right)\frac{2d}{2d+\nu}, \quad \bar{i} + \bar{k} = K\left(1 - \frac{d}{r}\right).$$

This result is quite intuitive; while the total population is maintained at the usual level that characterizes a homogeneous population dividing at rate r and dying at rate d, the cells are split into dividing and arrested subpopulations, and the fraction of cells in stage zero decreases as ν grows. Let us now calculate the *per capita* division and death rates at equilibrium, given by $r\bar{i}/(\bar{i}+\bar{k})$ and $d\bar{i}/(\bar{i}+\bar{k})$:

$$\bar{r}_x = \frac{r\nu}{2d+\nu}, \quad \bar{d}_x = \frac{d\nu}{2d+\nu}.$$

By the same logic, we have

$$\bar{r}_y = r, \quad \bar{d}_y = d,$$

since the dividing population is the same as the total population of mutants. The relative turnover parameter for the mutant population (see equation (1)) is then given by

$$\alpha = \frac{2d+\nu}{\nu}.$$

Since increasing d and decreasing ν increases α, it follows from results of Section 1 that as a consequence, the effect of negative selection will be stronger for larger death rates and smaller ν. This is what we observed in figure 2. We also observe that as $\nu \to \infty$, or as $d \to 0$, the turnover parameter $\alpha \to 1$, and the mutants will behave as neutral. The dependence on ν and d is quite intuitive. Decreasing ν and increasing d increases the percentage of stage zero cells and decreases the percentage of dividing cells at the equilibrium (for different reasons: small ν means that arrested cells remain arrested for longer, and large d depletes the dividing compartment). Because the dividing cells do all the division/death work, the per cell division and death rates of wild type cells decrease, thus increasing the difference between the wild type cells and mutants, and making mutants stand out more (i.e. increasing the turnover parameter α).

Finally, we note that exactly the same reasoning applies if we define W as in equation (13) (that is, cells divide without restriction until the whole space, N, is filled, and they do not divide once the population reaches size N), or use any other formulation of space limitation/competition that allows a stable solution. In this case, the total population will oscillate around its mean size, $\bar{N}$, and deaths will be balanced by divisions, that is, $di = rWi$. The expected increment of the wild type cells is then given by

$$\Delta k = 2rWi - \nu k = 2di - \nu(\bar{N} - i) = 0,$$

and therefore we have in steady state

$$\bar{i} = \bar{N}\frac{\nu}{2d+\nu}, \quad \bar{k} = \bar{N}\frac{2d}{2d+\nu}.$$

The rest of the argument remains unchanged.

2.3 The Moran process with an arresting state

One can also formulate the Moran process for the populations where the wild types enter stage zero upon divisions, and mutants do not. We characterize the states of the system by the numbers of active wild type cells and mutants, (i, j), with the number of arrested cell $k = N - i - j$. Let us start with a death-birth process, which proceeds as a sequence of updates, some of which are death-birth updates and some are transition (from stage zero to active wild type cells) events. Denote by $\mathcal{M}_{db} = d_x i + d_y j + \nu(N - i - j)$. Then the nonzero transition probabilities can be written as follows:

$$\text{w.t. dies, w.t. divides: } P_{(i,j)\to(i-2,j)} = \frac{d_x i}{\mathcal{M}_{db}} \frac{r_x(i-1)}{r_x(i-1) + r_y j}, \tag{17}$$

$$\text{w.t. dies, mutant divides: } P_{(i,j)\to(i-1,j+1)} = \frac{d_x i}{\mathcal{M}_{db}} \frac{r_y j}{r_x(i-1) + r_y j}, \tag{18}$$

$$\text{mutant dies, w.t. divides: } P_{(i,j)\to(i-1,j-1)} = \frac{d_y j}{\mathcal{M}_{db}} \frac{r_x i}{r_x i + r_y(j-1)}, \tag{19}$$

$$\text{mutant dies, mutant divides: } P_{(i,j)\to(i,j)} = \frac{d_y j}{\mathcal{M}_{db}} \frac{r_y(j-1)}{r_x i + r_y(j-1)}, \tag{20}$$

$$\text{arrested cell wakes up: } P_{(i,j)\to(i+1,j)} = \frac{\nu(N-i-j)}{\mathcal{M}_{db}}, \quad i + j > 1. \tag{21}$$

Solving the system

$$\rho_{(i,j)} = \sum_{0\le i'+j'\le N} P_{(i,j)\to(i',j')}\ \rho_{(i',j')}, \quad 0 \le i+j \le N, \quad 0 < j < N, \tag{22}$$

with $\rho_{(i,0)} = 0$ and $\rho_{(0,N)} = 1$, we obtain that

$$\rho_{(i,j)} = \frac{j}{N},$$

that is, the mutants behave as if they are neutral. This result goes against the intuition that we have developed by analyzing all the previous models. In particular, we observe that the probability of mutant fixation seems independent of ν, the rate of stage zero cells becoming active.

To see exactly why the mutants are neutral in this system, we note that, taking $d_x = d_y = d$, $r_x = r_y = r$, the probabilities of mutant increase and decrease are equal to each other:

$$P^{db}_{j\to j+1} = \frac{di}{\mathcal{M}_{db}} \frac{j}{(i-1)+j} = \tag{23}$$

$$P^{db}_{j\to j-1} = \frac{dj}{\mathcal{M}_{db}} \frac{i}{i+(j-1)}. \tag{24}$$

Before we turn to the birth-death formulation, we investigate what happens when $\nu = 0$. In this case, mutant fixation is impossible if there is at least one stage zero cell in the system initially. Setting $\nu = 0$ in the above algebraic system makes it underdetermined, such

that values $\rho_{(i,j)}$ with $i + j < N$ and $j \neq 0$ are left undefined. If we set them to zero, the probabilities of fixation in the absence of stage zero cells, $\rho_{(i,j)}$ with $i + j = N$, are obtained as small nontrivial values (fixation is these cases is only possible if wild type cells die out without ever dividing, which is possible but unlikely; the exact solution of this process is given in section 2.4). Note that in the case of death-birth Moran formulation of a system with stage zero, the limit of the solution as $\nu \to 0$ is not equal to the solution of the system where parameter $\nu = 0$ is used.

Next, consider the birth-death formulation of the Moran process. Defining $\mathcal{M}_{bd} = r_x i + r_y j + \nu(N - i - j)$, the list of events is given below:

$$\text{w.t. divides, w.t. dies: } P_{(i,j)\to(i-2,j)} = \frac{r_x i}{\mathcal{M}_{bd}} \frac{d_x(i-1)}{d_x(i-1) + d_y j}, \tag{25}$$

$$\text{w.t. divides, mutant dies: } P_{(i,j)\to(i-1,j-1)} = \frac{r_x i}{\mathcal{M}_{bd}} \frac{d_y j}{d_x(i-1) + d_y j}, \tag{26}$$

$$\text{mutant divides, w.t. dies: } P_{(i,j)\to(i-1,j+1)} = \frac{r_y j}{\mathcal{M}_{bd}} \frac{d_x i}{d_x i + d_y(j+1)}, \tag{27}$$

$$\text{mutant divides, mutant dies: } P_{(i,j)\to(i,j)} = \frac{r_y j}{\mathcal{M}_{bd}} \frac{d_y(j-1)}{d_x i + d_y(j+1)}, \tag{28}$$

$$\text{stage zero cell wakes up: } P_{(i,j)\to(i+1,j)} = \frac{\nu(N-i-j)}{\mathcal{M}_{bd}}, \quad i + j > 1. \tag{29}$$

In this case, solving the system for the fixation probabilities, we notice that the mutants experience negative selection. Figure 3 illustrates this by plotting the probability of mutant fixation (starting from one mutant cell and $N - 1$ active wild type cells) as a function of parameter ν, for several particular values of N. We can see that for all values of ν, the mutants are selected against. Incidentally, just as in the case of a death-birth process, the limit of the solution for the fixation probabilities as $\nu \to 0$ is not equal to the solution of the $\nu = 0$ system. The latter is underdetermined, and if all the probabilities of fixation starting from nonzero numbers of arrested cells are set to zero, it is given by a recursive relationship derived in section 2.4.

To explain the fact that in the birth-death process, the mutants are selected against, we again examine the probabilities of mutant increase and decrease, which are given by

$$P^{bd}_{j\to j+1} = \frac{rj}{\mathcal{M}_{bd}} \frac{i}{i + (j+1)} < \tag{30}$$

$$P^{bd}_{j\to j-1} = \frac{ri}{\mathcal{M}_{bd}} \frac{j}{(i-1) + j}. \tag{31}$$

We can see the subtle difference between the birth-death and death-birth formulations, which plays a significant role in the mutant fate. In the death-birth process, see equation (24), after the first event (cell death), the total number of cells competing for the chance to divide decreases by one, making the probabilities of mutant increase and decrease exactly the same, as they both mutants and active wild type cells have the same kinetic parameters and compete among each other for both death and divisions. In the birth-death formulation

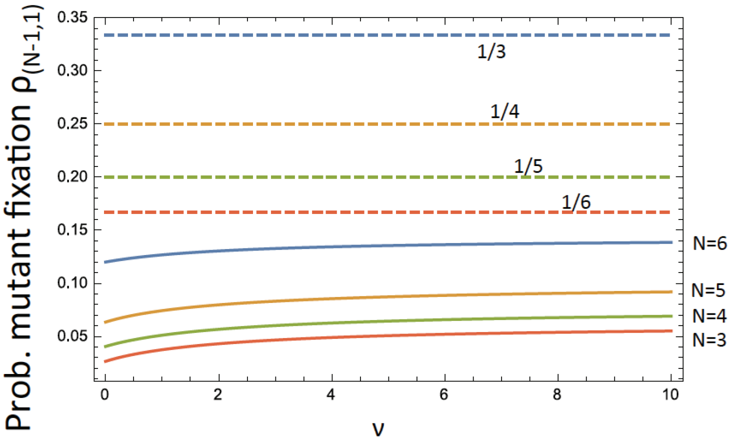

Figure 3: The birth-death formulation of the Moran process with stage zero. Mutant fixation probability, $\rho_{N-1,1}$, as a function of ν (the rate of transition back from the arrested state) is shown for several values of N. Dashed horizontal lines show the neutral fixation probability, $1/N$. We used $r = 1$.

(inequality 31), the first event (division) changes the active population size in a different way depending on what type divdes. If it is a mutant that divides, the population of active cells increases. If it is a wild type cell that divides, the population of active cells decreases. As a consequence, the second pair of events (division of a wild type followed by a death of a mutant) is more likely than the first one (division of a mutant followed by a death of a wild type). Therefore, mutants have a higher probability to decrease than to increase, and are thus selected against.

When we consider a contact process, events come in a random order (but balance each other). Death-birth pairs of events do not put any selective pressure on a mutant, while birth-death pairs select against mutants. The overall net effect is negative selection, exactly as we observed when studying the contact process.

2.4 The limit of $\nu \to 0$

In this limit, we can consider the Markov chain characterized by the number of mutants, j, with the number of wild types given by $N - j$, and any transition to a state with $i + j < N$ equivalent to the impossibility of mutant fixation. For simplicity, let us take $d_x = d_y$ and $r_x = r_y$. **For the death-birth formulation**, the following transitions are between nonzero probabilities of fixation:

$$P_{j\to j+1} = \frac{(N-j)j}{N(N-1)}, \quad P_{j\to j} = \frac{j(j-1)}{N(N-1)},$$

where the first transition is a death of wild type and division of a mutant, and the second one is a death and a division of a mutant. All other transitions result in a state with stage zero cells, which is absorbing and is characterized by an impossibility of mutant fixation. We have

$$\rho_j = P_{j\to j+1}\rho_{j+1} + P_{j\to j}\rho_j, \quad 1 \le j \le N-1, \quad \rho_N = 1.$$

Therefore, the resulting fixation probabilities are given by the recursion

$$\rho_N = 1, \quad \rho_j = \rho_{j+1}\frac{(N-j)j}{N(N-1)-j(j-1)}, \quad j = N-1, N-2, \ldots, 1.$$

For the birth-death formulation, the following transitions are between nonzero probabilities of fixation:

$$P_{j\to j+1} = \frac{j(N-j)}{N(N+1)}, \quad P_{j\to j} = \frac{j(j+1)}{N(N+1)},$$

where the first transition is a division of a mutant followed by a death of a wild type cell, and the second one is a division and a death of a mutant. All other transitions result in a state with stage zero cells, which is absorbing and is characterized by an impossibility of mutant fixation. We have

$$\rho_j = P_{j\to j+1}\rho_{j+1} + P_{j\to j}\rho_j, \quad 1 \le j \le N-1, \quad \rho_N = 1.$$

Therefore, the resulting fixation probabilities are given by the recursion

$$\rho_N = 1, \quad \rho_j = \rho_{j+1}\frac{(N-j)j}{N(N+1)-j(j+1)}, \quad j = N-1, N-2, \ldots, 1.$$

References

[1] Kaveh, K., Komarova, N. L., & Kohandel, M. (2015). The duality of spatial death?birth and birth?death processes and limitations of the isothermal theorem. Royal Society open science, 2(4), 140465.
